# Supplementary material for: Weaving a cocoon on the way to aging transcendence: Grounded theory study on aging perception during menopause transition
Source: PLoS One. 2022 Nov 1;17(11):e0276797. doi: 10.1371/journal.pone.0276797 (PMC9624393; doi:10.1371/journal.pone.0276797)
Supplement: S1 Table — (DOC) [file pone.0276797.s001.doc]

| **Subject** | **Initial questions** | **Probing questions1** | **Probing questions2** |
| --- | --- | --- | --- |
| I am PhD student in gerontology and I am working on my thesis, which is related to menopause transition women. If you are satisfied, I would like you to go back to the time through menopause and think about your experience and feelings in relation to the questions. Please, Tell me about: | | | |
| **Open Questions** | What was the first feeling you had when you found out were going through menopause? Explain? | Since when do you feel old? Explain? | Why do you think menopause is the beginning of aging? |
|  | Describe your age group? How did you get to this stage? | What did you experience and feel when you say: "I am old"? |  |
|  | How did you experience reproductive aging? | What effects has reproductive aging had on your sense of aging? | How do society's beliefs and faiths related to fertility affect your sense of aging? |
|  | What do you think changed for you when you went through menopause? | How did these changes in your body make you feel old? | What did you do after the physical changes related to aging? How? What did you outcomes get? |
|  |  | How did these changes in your sexual make you feel old? | What did you do after the sexual changes related to aging? How? What did you outcomes get? |
|  |  | How did these changes in your Spirit, soul, and mood make you feel old? | What did you do after your psychological changes related to aging? How? What did you outcomes get? |
|  |  | How did these changes in your activities and performance make you feel old? | What did you do after functional changes related to aging? How? What did you outcomes get? |
|  |  | How did family and community hints affect your sense of aging? | What was your reaction to the behavior of family and people around you in relation to old age? How? What did you outcomes get? |
|  |  | How do the beliefs of society affect your sense of aging? | What is your reaction to these religious and beliefs about the sense of old age? How? What did you outcomes get? |
| **End question** | Finally, If I ask you: how were your experience of menopause along with aging, what would you say? | | |

**Interview Guide**
