# Supplementary material for: Weaving a cocoon on the way to aging transcendence: Grounded theory study on aging perception during menopause transition
Source: PLoS One. 2022 Nov 1;17(11):e0276797. doi: 10.1371/journal.pone.0276797 (PMC9624393; doi:10.1371/journal.pone.0276797)
Supplement: S2 Table — (DOC) [file pone.0276797.s002.doc]

| موضوع | سوالات اولیه  **راهنمای مصاحبه** | سوالات پیگیری و کاوشی1 | سوالات پیگیری و کاوشی2 |
| --- | --- | --- | --- |
| معرفی | اینجانب دانشجوی دکتری تخصصی سالمند شناسی هستم و دارم روی پایان نامه ام که در ارتباط با خانم های یائسه هست کار می کنم . مایل هستم در صورت رضایت ، شما برگردید به عقب از زمانی که یائسه شدین تجربه و حس خودتون رو در ارتباط با سوالاتی که می پرسم فکر کنید و توضیح بدین | | |
| سوالات باز | از وقتی فهمیدین دارین یائسه میشید اولین حسی که داشتین چی بود؟ توضیح بدین؟ | حس می کنی از چه زمانی پیر شدی؟ توضیح بدین؟ | چرا فکر می کنید یائسگی شروع پیری بوده؟ |
|  | گروه سنی خودتون را توصیف کنید؟ به نظرتون چطوری وارد این مرحله شدید؟ | چه چیزی تجربه و حس کردین که میگین "من پیر هستم"؟ |  |
|  | پیری باروری را چطور تجربه کردید؟ | پیری باروری چه تأثیراتی بر حس پیری شما داشته است؟ | اعتقادات و باورهای جامعه در ارتباط با باروری به چه صورت روی حس پیری شما تأثیرداشته؟ |
|  | زمانی که یائسه شدین به نظرتون چه چیزی هایی براتون تغییر کرده؟ | این تغییرات در جسم شما به چه صورت باعث حس پیری شد | چه کارایی بعد از تغییرات جسمی در ارتباط با پیری انجام دادین؟به چه صورت؟به چه نتیجه ای رسیدین؟ |
|  |  | این تغییرات در ارتباط جنسی شما به چه صورت باعث حس پیری شد؟ | چه کارایی بعد از تغییرات جنسی در ارتباط با پیری انجام دادین؟به چه صورت؟به چه نتیجه ای رسیدین؟ |
|  |  | این تغییرات در روحیه و روان شما به چه صورت باعث حس پیری شد | چه کارایی بعد از تغییرات روحی روانی خودتون در ارتباط با پیری انجام دادین؟به چه صورت؟ به چه نتیجه ای رسیدین؟ |
|  |  | این تغییرات در فعالیت ها و عملکرد شما به چه صورت باعث حس پیری شد | چه کارایی بعد از تغییرات فعالیتی در ارتباط با پیری انجام دادین؟به چه صورت؟ به چه نتیجه ای رسیدین؟ |
|  |  | پیام های خانواده و جامعه به چه صورت روی حس پیری شما تأثیرداشته؟ | عکس العمل شما نسبت به رفتار خانواده و اطرافیان در ارتباط با پیری چی بود؟ به چه نتیجه ای رسیدین؟ |
|  |  | اعتقادات و باورهای جامعه به چه صورت روی حس پیری شما تأثیرداشته؟ | عکس العمل شما نسبت به این اعتقادات مذهبی و باورها نسبت به حس پیری چیه؟ به چه نتیجه ای رسیدین؟ |
| سوال پایانی | اگر ازت بپرسم "در کل تجربه یائسگی همراه با پیری چطور بوده " چی میگید؟ | | |
